# Effect of herbal feed additives on milk performance and health status of dairy goats

**DOI:** 10.2478/jvetres-2025-0021

**Published:** 2025-04-04

**Authors:** Jacek Antoni Wójtowski, Jan Pikul, Przemysław Mikołajczak, Michał Czopowicz, Jarosław Kaba, Joanna Foksowicz-Flaczyk, Ireneusz Antkowiak, Jarosław Pytlewski, Maria Markiewicz-Kęszycka, Daniel Stanisławski

**Affiliations:** 1 Department of Animal Breeding and Product Quality Assessment, Faculty of Veterinary Medicine and Animal Science, Poznań University of Life Science, 62-002 Suchy Las, Poland; 2 Department of Dairy and Process Engineering, Faculty of Food Science and Nutrition, Poznań University of Life Sciences, 60-624 Poznań, Poland; 3 Department of Pharmacology, Poznań University of Medical Sciences, 60-806 Poznań, Poland; 4 Division of Veterinary Epidemiology and Economics, Institute of Veterinary Medicine, Warsaw University of Life Sciences-SGGW, 02-776 Warsaw, Poland; 5 Department of Innovative Biomaterials and Nanotechnologies, Institute of Natural Fibres and Medicinal Plants, 60-630 Poznań, Poland; 6 School of Agriculture and Food Science, University College Dublin, Belfield, Dublin 4, Ireland; 7 Computer Laboratory, Poznań University of Life Sciences, 60-637 Poznań, Poland

**Keywords:** dairy goats, herbal feed additives, milk, haematological parameters, acute phase proteins

## Abstract

**Introduction:**

Interest is increasing in natural feed additives that improve animal health, raise farming productivity and enhance the quality of animal products. These additives, especially polyphenols, are biologically active chemical compounds found in plants.

**Material and Methods:**

Sixty dairy goats were randomly assigned to five feeding groups of 12 animals each. Over 16 weeks, the animals received a polyherbal supplement containing seven or nine herb species at 20 or 40 g/animal/day, along with pelleted concentrate feed. The health status of the animals was assessed based on the concentration of acute phase haptoglobin proteins and serum amyloid A in blood serum.

**Results:**

A statistically significant positive effect of the herbal mixtures was found on the percentage of milk fat and fat : protein ratio (P-value < 0.05). The time of test-day milking and milk sampling had a significant impact on the level of all examined milk parameters (P-value < 0.001). Moreover, milk yield and fat-corrected milk yield were significantly affected by a feeding group × time of test-day milking and milk sampling interaction, influencing the level of examined parameters.

**Conclusion:**

The use of herbal supplements in the diet of dairy goats did not negatively affect the goats’ milk production – neither the yield nor composition. A positive effect of the administered multi-herbal mixtures was found on % fat concentration and fat : protein ratio in milk.

## Introduction

After many years when antibiotics were used in animal feeds to improve animal performance, the European Union banned their use (15). Interest in natural feed additives that improve animal health, raise livestock farming productivity and enhance the quality of animal products is therefore increasing among farmers and the scientific community (19). Feed additives containing phytochemicals are a promising alternative to antibiotics. These additives, especially polyphenols, are biologically active chemical compounds found in plants which provide health benefits beyond those attributed to macronutrients and micronutrients (10). Phytochemicals accumulate in different parts of the plants: the roots, stems, leaves, flowers, fruits or seeds. Medicinal plants, commonly known as herbs, are particularly rich in these compounds (6), and when used in the diet of ruminants may enhance feed taste and stimulate appetite. As regulators of digestive functions, they influence the motility of the digestive tract and the secretion of digestive juices, reduce the occurrence of diarrhoea, and regulate the pH of the digestive tract, ruminal fermentation and bacterial diversity (11). They can also have a protective effect as metabolism regulators and a beneficial one on animal product quality (30). Some herbs have anabolic, anti-stress and strengthening properties, can even counteract the negative impact of anti-nutritional substances in feed, and by demonstration in lactating cows, they often increased milk production (18). The bioactive properties of polyphenols also play a significant role in preventing many chronic diseases. The largest group of naturally occurring polyphenols are flavonoids, which include apigenin (AP). Chlorogenic acid (CA) is a polyphenolic compound produced by plants through the shikimic acid pathway during aerobic respiration (13). Both these compounds have antioxidant, anti-inflammatory and anticancer properties, can inhibit platelet aggregation and can reduce the level of low-density lipoproteins in plasma and cell proliferation (16). Apigenin occurs mainly in the form of glucosides in herbs, including chamomile and thyme, and in vegetables and fruits (23).

Because of the biological activity of AP and CA, their concentration level in plants as well as bioavailability for humans and animals are of great importance. Karaźniewicz-Łada *et al*. (13) observed significantly higher concentrations of AP and CA in the serum of dairy goats fed a diet supplemented with 40g/day/animal of a polyherbal additive compared to AP and CA concentrations in the serum of an unsupplemented control group. These results indicate the positive nutritional effect that may improve goats’ health and impart health benefits to human consumers of dairy goat products (13). Other studies conducted on goats have shown the positive effect of nutritional herbal additives on milk production and composition and on intestinal lactic acid bacteria count (8). Kholif *et al*. (15) demonstrated that a supplement of 10g/goat/day of rosemary and lemongrass had a significant positive effect on daily milk yield and milk chemical composition (P-value < 0.001). The authors also demonstrated that the fat in the goats’ milk contained a significantly higher proportion of unsaturated fatty acids and conjugated linoleic acid and a lower proportion of total saturated fatty acids (P-value < 0.05). Most studies did not show any negative effects of herbal supplements on the health status of animals (9). However, because of the wide variation in the composition and dosage of herbal supplements for farm animal feed, assessing their impact on animal health is exigent. The aim of this study was to investigate the impact of multiherbal feed additives administered to dairy goats in the amount of 20 or 40 g/animal/day on the production and chemical composition of milk, as well as on the health status of the experimental animals. The hypothesis was tested that herbal feed additives used in goat diets positively affect the animals’ health, productivity and milk composition. To the best of the authors’ knowledge, very little scientific literature comprehensively discusses this issue, which is nevertheless of great scientific and practical importance.

## Material and Methods

### Animals

The 16-week experiment was conducted on 60 Polish White Improved goats on a dairy goat farm in Western Poland. The goats were cared for in accordance with the guidelines of the Local Ethical Commission for Investigations on Animals (permit No. 57/2020) and the National Ethical Commission for Animal Research (under the Polish Ministry of Science and Higher Education). All goats were healthy and the herd was under permanent veterinary monitoring. Standard parasitological tests confirmed that the experimental animals were free from parasitic infections. Veterinary examination also confirmed that animals did not have clinical or sub-clinical mastitis. The somatic cell count (SCC) measured before the experiment did not exceed 800 × 10^3^/mL. The body weight of the experimental animals was in the range of 56–60 kg, and all the goats were between 20 and 30 months old and in their second lactation. At the beginning of the experiment, the animals were at 28.1 ± 2.7 days in milk. The animals were randomly divided into five feeding groups of 12 goats, in which besides total mixed ration they were given pellet concentrates into which herbal supplements had been mixed. One group was supplemented with a mixture of seven herbs and each goat received 20 g daily; these animals were designated the 7/20 g group. Another was supplemented with the same mixture and an animal received 40 g daily, becoming the 7/40 g group. Another was offered a mixture of nine herbs in a daily 20 g supplement and was the 9/20 g group. The final experimental group’s goats each ate 40 g of the mixture of nine herbs every day and were the 9/40 g group. Animals from the control group (CTR) did not receive any herbal supplement. Each group of goats was kept in a separate pen. The experimental factors were two: the herbal mixture was prepared from seven or from nine herbs, and was given to animals at 20 or 40 g per day.

### Herbal supplements

The herbal mix composed of seven herbs comprised common nettle, common agrimony, caraway, coriander, fenugreek, plantain and purple willow. The mix of nine herbs included different amounts of three of the herbs that were also used in mix of seven herbs, *i.e*. common nettle, common agrimony and coriander, and additionally included fennel, peppermint, chamomile, milk thistle and thyme (Table 1).

**Table 1.** Components of the herbal mixtures with which the studied dairy goats were supplemented

Herbal mixture	Herb and proportion in the mixture (% dry matter)
Composed of seven herbs	Common nettle *Urtica dioica* L. (leaves): 20%; Common agrimony *Agrimonia eupatoria* (dried flowering shoot tips) 10%; Caraway *Carum carvi* (fruit) 10%; Coriander *Coriandrum sativum* (fruit) 15%; Fenugreek *Trigonella foenum-graecum* L. (seeds) 20%; Plantain *Plantago lanceolata* L. (herb) 20%; Purple willow *Salix purpurea* (bark) 5%.
Composed of nine herbs	Common nettle *Urtica dioica* L. (leaves): 10%; Common agrimony *Agrimonia eupatoria* (herb – dried flowering shoot tips) 10%; Fennel *Foeniculum vulgare* (fruit) 15%; Coriander *Coriandrum sativum* (fruit) 15%; Fenugreek *Trigonella foenum-graecum* L. (seeds) 10%; Peppermint *Mentha piperita* (leaves) 16%; Chamomile *Matricaria chamomilla* L. (flower clusters) 4%; Milk thistle *Silybum marianum* (endosperm) 4%; Thyme *Thymus vulgaris* (leaves) 16%.

### Animal nutrition

The goats’ diet adhered to the nutritional requirements for lactating goats according to the Institute National de la Recherche Agronomique (INRA) recommendations from 2018: 2.12 units for milk production and 185 g of protein truly digestible in the small intestine to obtain an assumed milk yield of 3.0 kg with 3.8% fat. The animals were fed a total mixed ration once daily.

The experimental concentrate with the herbal mix was offered to animals separately, during milking in a milking parlour. It included wheat bran, triticale, rapeseed meal, sunflower meal and herbal supplements. The dose of additives used and the detailed composition of the experimental concentrates are presented in the work of Foksowicz-Flaczyk *et al*. (8). Goats had unlimited access to water and a mineral salt lick. The composition of the diet offered to the dairy goats is presented in Table 2.

**Table 2.** Composition of the diet offered to the studied dairy goats

	Component	% diet dry matter
Incorporated in total mixed ration	Maize silage	15.6
	Grass hay silage	21.6
	Brewers’ grain silage	7.8
	Concentrate mixture (triticale, corn grain, lupine grain, rapeseed meal and sunflower meal)	26.4
	Meadow hay	10.3
	Dried sugar beet pulp	4.4
	Barley straw	3.3
Not in total mixed ration	Experimental concentrate	10.6
	Chemical composition	g/kg dry matter
	Organic matter	451
	Crude protein	163
	Acid detergent fibre	267
	Neutral detergent fibre	401

### Daily milk yield evaluation, and sampling and chemical analyses of milk

The goats were milked twice daily in a milking parlour at 5:30 a.m. and 5:30 p.m. Milking was performed at a vacuum pressure of 42 kPa and pulsation rate of 90 pulses/min. Udders and teats were disinfected before and after milking (26). Milk yield was assessed on the basis of four twice-daily test-day milkings performed in the 4^th^, 8^th^, 12^th^ and 16^th^ weeks of the experiment. Milk yield measurement from the morning and afternoon milkings was recorded using a Sylco I-Meter (Sylco Hellas, Thessaloniki, Greece) with an accuracy of 0.01 kg. Eight individual milk samples were also taken from each animal during test-day milkings as one morning and one afternoon sample each time, making 480 samples in total. Milk composition (fat, protein, casein, lactose, total solids (TS), solids not fat, urea, freezing point depression (FPD) and acidity (Ph)) was analysed with a MilkoScan FT analyser (FOSS Analytical, Hillerød, Denmark) previously calibrated for goats’ milk. Milk SCC levels were determined with the Bactocount IMCm flow cytometry instrument (Bentley, Chaska, MN, USA).

### Blood collection

Blood samples were collected in the last (16^th^) week of the experiment, before the afternoon test-day milking. The blood was collected from the jugular vein in 10-mL dry tubes. The tubes were left at room temperature overnight to allow clot formation, then centrifuged at 3,000 rpm for 10 min. The serum was stored in 2-mL Eppendorf vials at –20°C until testing.

### Analyses of haematological and biochemical blood profiles

Haematological analyses were performed in a commercial veterinary laboratory using an Abacus Hematology analyser (Diatron MI, Budapest, Hungary), and biochemical analyses were undertaken with a BS-800 automatic photometric clinical chemistry analyser (Mindray Bio-Medical Electronics, Shenzhen, China). Haematology analysis included the determination of the red blood cell (erythrocyte) count (RBC) in tera/L (T/L), haemoglobin concentration (HGB) in g/dL, haematocrit percentage (HCT), platelet (thrombocyte) count (PLT) in giga/L (G/L) and white blood cell (leukocyte) count (WBC) in G/L. Three RBC indices were calculated: the mean corpuscular volume (MCV) in fL derived by dividing HCT by RBC, the mean corpuscular haemoglobin (MCH) in pg found by dividing HGB by RBC, and the mean corpuscular haemoglobin concentration (MCHC) in g/dL as the result of dividing HGB by HCT. The differential white blood cell count was performed on thin, high-quality peripheral blood smears stained with the May-Grünwald–Giemsa reagent. The smears were examined in a Primo Star light microscope (Carl Zeiss, Oberkochen, Germany) under 100× magnification by a qualified technician. One hundred nucleated cells were classified into six subpopulations: band neutrophils, segmented neutrophils, eosinophils, basophils, monocytes and lymphocytes. Their counts were calculated by multiplying WBC by the proportion of each subpopulation and were expressed in G/L. Biochemistry analysis included concentration of triacylglycerols (triglycerides), total cholesterol, LDL cholesterol, HDL cholesterol and glucose, all noted in g/dL.

### Acute phase protein measurements

Haptoglobin (Hp) concentration was measured using a colourimetric assay (Tridelta PHASE Haptoglobin Assay, Tridelta Development, Maynooth, Ireland) and recorded in g/L, and the serum amyloid A (SAA) concentration was gauged using an immunoenzymatic Phase SAA Multispecies Assay (Tridelta Development) with mg/L as its unit. Both assays were performed according to the manufacturer’s manual with original control samples (Haptoglobin Control and SAA Control; Tridelta Development) optimised for these assays. Serum samples were diluted 1:2 in the Hp assay and 1:100 in the SAA ELISA. The limits of detection of the assays were 0.005 g/L and 0.3 mg/L, respectively. Intra- and inter-plate precision (repeatability) expressed as the coefficient of variation (CV%) were respectively 6.3% and 5.7% in the Hp assay, and 5.0% and 11.4% in the SAA ELISA. The optical density of the samples was read using an Epoch Microplate Spectrophotometer (BioTek, Winooski, VT, USA) at the wavelength of 630 nm to find the Hp concentration or 450 nm to obtain the SAA concentration.

### Statistical analysis

Data (milk yield, milk composition and SCC) were analysed using the PROC MIXED procedure in SAS/STAT v. 9.4 (SAS Institute, Cary, NC, USA). The lowest Akaike information Criterion was used to determine the appropriate structure within subject covariance, and the compound symmetry was selected accordingly.

Data were analysed as repeated measures (the random effect of an individual goat) using the following model:

$${\text{Y}}_{\text{ijk}}{\;=\;\mu \;+\;\text{g}}_{\text{i}}{\;+\;\text{t}}_{\text{j}}\;+\;(\text{g}\times \;{\text{t)}}_{\text{ij}}{\;+\;\text{e}}_{\text{ijk}}$$

where Y_ijk_ is the dependent variable, μ is the overall mean, gi is the fixed effect of the feeding group (i = 1, 2, 3, 4, 5), t_j_ is the fixed effect of the time of test-day milking and milk sampling (j = 1, 2, 3, 4), (g × t)_ij_ is the interaction between the i^th^ feeding group and the j^th^ time of test-day milking and milk sampling and eijk is the residual error.

Detailed comparisons of object means were performed using Tukey’s test. When differences were detected in terms of treatment or interactions of treatment with time, separation of means was conducted using Tukey’s adjustment for the probability. The statistical significance was considered to be P-value ≤ 0.05.

In order to assess the significance of the impact of the feeding group type (the herbal mixture of seven herbs or the one of nine herbs, and its dose at 20 or at 40 g per day) on the haematological and biochemical blood parameters, an analysis of variance was used in the PROC GLM (general linear model) procedure in SAS/STAT v. 9.4:

$${\text{Y}}_{\text{ij}}{\;=\;\mu \;=\;\text{g}}_{\text{i}}{\;+\;\text{e}}_{\text{ij}}$$

where Y_ij_ is the phenotypic value of the analysed trait, μ is the population average, g_i_ is the fixed effect of the feeding group (I = 1, 2, 3, 4, 5) and e_ij_ is the random error.

Detailed comparisons of object means were again performed using Tukey’s test (P-value = 0.05).

The PROC UNIVARIATE procedure in SAS/STAT v. 9.4 was used to assess the normality of distribution. The data relating to SCC in the milk were log10-transformed for analyses.

Fat-corrected milk yield at 3.5% of fat (kg/day) was calculated according to Pulina *et al*. (22) using the following equation:

Fat-corrected milk 3.5% (FCM) = daily milk yield × (0.634 + 0.1046 × fat content).

The analysis of morphological and biochemical parameters of blood, as well as calculation of their Spearman’s correlation with acute phase proteins (APPs), was performed in SAS/STAT v. 9.4. Concentrations of APPs were compared between groups of goats using the Kruskal–Wallis H test with Dunn’s post-hoc test. Proportions of goats with APPs above the reference intervals (RIs) were compared between groups with the maximum likelihood G-test. All tests were two-tailed. A significance level (α) was set at 0.05, and a Bonferroni correction was applied for multiple comparisons. The analysis was performed in TIBCO Statistica 13.3 (TIBCO Software, Palo Alto, CA, USA). The Hp and SAA RIs were 0.399–1.242 g/L and 0.435–2.050 mg/L, respectively, based on data published by Heller and Johns (12).

## Results

The effects of herbal supplements (feeding group) and time of test-day milking and milk sampling on milk yield, milk composition, processing properties and SCC are presented in Table 3. The effect of the feeding group was statistically significant on fat percentage in milk (P-value = 0.0323). In the milk of experimental goats, this percentage was significantly higher than in the milk of the CTR group (P-value < 0.05). Milk obtained from the 7/20 g group had the highest fat level – 4.32% – which differed statistically significantly (P-value < 0.05). Moreover, a significant effect of herbal supplements was also noted for fat : protein ratio (FPR) and pH acidity (P-values respectively 0.0026 and 0.0074). The time of test-day milking and milk sampling had a significant impact on the levels of all studied milk parameters (P-value = 0.0001). Generally, the basic composition of milk (percentage of fat, protein, casein and dry matter) in the 16^th^ (and last) week of the experiment was characterised by a higher level of values compared to the level recorded during the first test-day milking (week 4).

**Table 3.** The influence of herbal supplement dietary group and lactation stage (time) on dairy goat milk yield, composition, some processing parameters and somatic cell count (SCC)

Parameter	Mean	Group					Time (week)				SE	Group	Time	Group × Time
		7/20 g	7/40 g	9/20 g	9/40 g	CTR	4	8	12	16			P-value	
Milk yield (kg/d)	2.75	2.61	2.63	2.69	2.93	2.89	2.93^a^	2.96^a^	2.80^a^	2.31^b^	0.05	0.6402	0.0001	0.0003
Fat corrected milk (kg/d)	2.90	2.80	2.83	2.83	3.08	2.95	3.02^a^	3.32^b^	2.79^d^	2.46^c^	0.05	0.8030	0.0001	0.0037
Fat (%)	4.06	4.32^a^	4.19^ab^	3.99^ab^	4.10^ab^	3.68^b^	3.84^a^	4.68^b^	3.53^d^	4.17^c^	0.05	0.0323	0.0001	0.3906
Protein (%)	3.38	3.29	3.45	3.35	3.48	3.32	3.37^a^	3.55^b^	3.16^c^	3.44^a^	0.02	0.3395	0.0001	0.2085
Lactose (%)	4.39	4.32	4.41	4.38	4.38	4.46	4.48^a^	4.33^b^	4.39^b^	4.36^b^	0.01	0.2027	0.0001	0.7794
Total solids (%)	12.66	12.73	12.90	12.55	12.82	12.28	12.59^a^	13.42^b^	11.91^c^	12.72^a^	0.07	0.3309	0.0001	0.5426
Solids not fat (%)	8.60	8.41	8.71	8.57	8.72	8.60	8.74^a^	8.74^a^	8.37^c^	8.55^b^	0.03	0.2628	0.0001	0.6030
fat : protein ratio	1.20	1.31^a^	1.22^a^	1.19^b^	1.17^b^	1.11^b^	1.14^a^	1.32^b^	1.12^a^	1.21^c^	0.01	0.0026	0.0001	0.3325
FPD (°C)	0.563	0.563	0.561	0.563	0.563	0.564	0.563^ab^	0.560^a^	0.565^c^	0.563^b^	0.001	0.7198	0.0031	0.2553
Milk urea (mg/L)	316	299	317	318	316	330	313^a^	358^b^	275^c^	317^a^	3	0.0628	0.0001	0.3474
Casein (%)	2.55	2.47	2.62	2.54	2.64	2.51	2.56^a^	2.69^b^	2.39	2.58^a^	0.02	0.3452	0.0001	0.2470
Acidity pH	6.60	6.59^ab^	6.59^ab^	6.58^b^	6.57^b^	6.67^a^	6.65^a^	6.60^b^	6.54^d^	6.61^bc^	0.01	0.0074	0.0001	0.0490
SCC (log10 cell/mL)	5.72	5.70	5.70	5.72	5.73	5.72	5.72^a^	5.76^a^	5.62^b^	5.77^a^	0.01	0.9313	0.0001	0.2634

1 7/20 g and 7/40 g – groups supplemented with seven herbs at 20 or 40 g/animal/day; 9/20 g and 9/40 g – groups supplemented with nine herbs at 20 or 40g/animal/day; CTR – control group; FPD – freezing point depression; SCC – somatic cell count;

a,b – values within a row with different superscript letters differ significantly at P-value ≤ 0.05

At the same time, milk yield and fat-corrected milk yield were significantly influenced by the group × time interaction, with P-values of 0.0003 and 0.0037, respectively. This indicates the level variation of a given factor over time in particular feeding groups. The reaction of the animals to the herbal supplement varied. For example, animals from the 7/20 g group experienced a gradual decrease in milk yield from the beginning to the end of the experiment. Milk production in the CTR group also had a downward trend during the first four weeks. However, in the period from the 8^th^ to the 12^th^ week of the experiment, we observed a reverse trend with yields reaching 3.15 kg of milk per day. The effect of the group × time interaction on milk acidity was significantly lower (P-value = 0.0490). The pH values of milk from individual nutritional groups obtained at different time intervals were typical of those of good, regular-quality goats’ milk.

Table 4 shows the mean values of haematological and haematochemical parameters for all dietary groups studied in this experiment. An Anderson–Darling normality test proved that the data were normally distributed (P-value > 0.05) with a Gaussian distribution, and no outlier values were found. None of the blood samples collected contained clots or underwent haemolysis. No effect of dietary group was found on RBC, HGB, MCV, MCH, MCHC, PLT, red blood cell distribution width coefficient of variation (RDW-CV) or WBC (P > 0.05). Mixture and dose did not affect any haematological parameter except HCT. The lowest HCT value was found in goats from the 9/20 g group and this was statistically significantly lower (P-value < 0.05); it was similar to that found in goats from the 7/40 g group, which was, however, not significantly lower (P-value > 0.05). It is worth emphasising that the HCT in the blood of goats from all feeding groups was within the reference range given by Aiello and Moses (1). The diet had no effect on the WBC. The proportions of neutrophils, lymphocytes, monocytes, eosinophils and basophils were also similar for all experimental groups (P-value > 0.05). The effect of the feeding group had a statistically significant impact only on one biochemical parameter – triglycerides (P-value ≤ 0.05). The lowest triglyceride concentration (15.67 mg/dL) was found in the blood serum of goats from the 9/40 g group and the highest concentration (22.83 mg/dL) was reported for goats from the 9/20 g group (P-value ≤ 0.05) (Table 4).

**Table 4.** Haematological and biochemical parameters of blood in the studied herbally supplemented dietary groups of dairy goats

	Mean	Group					SE	P-value	Reference range
		7/20 g	7/40 g	9/20 g	9/40 g	CTR			
Haematological parameters									
RBC (T/L)	12.38	12.37	12.06	11.82	12.98	12.66	0.20	0.3715	8–18*
HGB (g/dL)	9.32	9.28	9.18	9.05	9.46	9.65	0.11	0.4895	8–12*
HCT (%)	24.10	24.88^a^	23.67^ab^	22.68^b^	24.37^a^	24.89^a^	0.27	0.0402	22–28*
MCV (fl)	19.96	20.24	20.08	19.98	19.81	19.69	0.18	0.8840	16–25*
MCH (p/g)	7.63	7.54	7.62	7.70	7.68	7.63	0.07	0.9653	5.2–8*
MCHC (g/dL)	38.01	37.34	38.75	38.55	38.30	37.10	0.41	0.6479	30–36*
PLT (G/L)	305.20	308.00	279.17	330.00	311.25	297.58	8.49	0.4335	160–490**
RDW-CV %	28.10	27.79	27.95	27.58	28.55	28.64	0.18	0.2546	
WBC (G/L)	11.27	10.40	10.73	11.86	11.47	11.88	0.34	0.5573	4–13*
Neutrophils (%)	56.11	54.27	58.84	57.02	54.92	55.51	1.45	0.8738	30–48*
Neutrophils (G/L)	6.40	5.69	6.32	6.84	6.47	6.68	0.28	0.7514	
Lymphocyte (%)	38.25	38.85	34.71	37.68	40.47	39.55	1.39	0.7362	50–70*
Lymphocyte (G/L)	4.24	3.97	3.72	4.39	4.48	4.63	0.17	0.4408	
Monocyte (%)	1.54	1.90	1.55	1.22	1.51	1.53	0.13	0.5782	0–4*
Monocyte (G/L)	0.17	0.20	0.17	0.14	0.15	0.18	0.02	0.8211	
Eosinophils (%)	3.43	4.09	4.08	3.30	3.08	2.60	0.31	0.4863	1–8*
Eosinophils G/L)	0.37	0.45	0.43	0.39	0.28	0.29	0.04	0.4659	
Basophils (%)	0.81	0.90	0.76	0.79	0.78	0.82	0.04	0.8006	0–1*
Biochemical parameters									
Cholesterol (mg/dL)	81.17	75.92	85.33	80.75	80.08	83.75	2.49	0.8001	80–115**
Triglycerydes (mg/dL)	19.35	18.00^ab^	20.75^ab^	22.83^a^	15.67^b^	19.50^ab^	0.73	0.0195	16–56**
LDL Cholesterol (mg/dL)	24.95	23.53	24.18	26.64	23.41	26.98	1.11	0.7569	21–58**
HDL Cholesterol (mg/dL)	61.30	57.09	66.57	57.99	61.72	63.15	2.05	0.5956	48–83**
Glucose (mg/dL)	51.50	49.42	50.75	55.00	52.42	49.92	0.91	0.2969	50–75*

1 7/20 g and 7/40 g – groups supplemented with seven herbs at 20 or 40 g/animal/day; 9/20 g and 9/40 g – groups supplemented with nine herbs at 20 or 40 g/animal/day; CTR – control group; SE – standard error; RBC – red blood cell count; HGB – haemoglobin concentration; HCT – haematocrit; MCV – mean corpuscular volume; MCH – mean corpuscular haemoglobin; MCHC – mean corpuscular haemoglobin concentration; PLT – platelet count; RDW-CV – red blood cell distribution width coefficient of variation; WBC – white blood cell count; HDL – high-density lipoprotein; LDL – low-density lipoprotein. Values within a row with different superscript letters differ significantly at P-value ≤ 0.05. Reference range

* – according to Aiello and Moses (1);

** – according to Dutra *et al*. (7)

Data for APPs in the blood plasma of all five groups of goats are presented in Table 5. Because the distribution of Hp and SAA was significantly right-hand asymmetric (coefficients of asymmetry = 4.01 and 6.02, respectively) their concentrations were presented as the median, interquartile range and range. No significant differences in SAA concentration between the five groups were found (P-value = 0.195). Although the omnibus Kruskal–Wallis H test indicated significant difference between groups in Hp (P = 0.010), the post-hoc Dunn’s test did not confirm any significant difference in pair-wise comparisons – only Hp in the 7/20 g group was close to significantly higher than Hp in the control group.

**Table 5.** Acute-phase proteins in the blood plasma of the studied herbally supplemented dietary groups of dairy goats

Group	Hp (g/L)	SAA (mg/L)
7/20 g	Median = 0.325	Median = 0
	IQR = 0.298–0.358	IQR = 0–0
	Range = 0.270–0.847	Range= 0–0
7/40 g	Median = 0.278	Median = 0
	IQR = 0.234–0.439	IQR = 0–1.890
	Range = 0.156–0.518	Range = 0–58.21
9/20 g	Median = 0.220	Median = 0
	IQR = 0.203–0.259	IQR = 0–0
	Range = 0.127–0.359	Range = 0–2.36
9/40 g	Median = 0.356	Median = 0
	IQR = 0.244–0.591	IQR = 0–0.009
	Range = 0.130–1.734	Range = 0–27.16
CTR	Median = 0.138	Median = 0
	IQR = 0.100–0.306	IQR = 0–1.714
	Range = 0.081–3.097	Range = 0–197.6
Kruskal–Wallis H test P-value	0.010	0.195
Comparisons between control group (CTR) and the four other groups		
Group	Dunn’s test P-value:	
7/20 g	0.052	-
7/40 g	0.456	-
9/20 g	0.999	-
9/40 g	0.103	-

1 Hp – haptoglobin; SAA – serum amyloid A; 7/20 g and 7/40 g – groups supplemented with seven herbs at 20 or 40 g/animal/day; 9/20 g and 9/40 g – groups supplemented with nine herbs at 20 or 40 g/animal/day; IQR – interquartile range; Range – minimum–maximum

The numbers of goats with APPs > RIs (12) are shown in Table 6. Haptoglobin and SAA were concurrently elevated >RIs in 3 out of 60 goats (5%), only SAA was elevated >RI in 6 goats (10%), and both APPs were normal in 51 goats (85%). Although a greater number of goats with increased Hp and SAA levels was found in the control group of animals, there was no significant difference in the proportion of goats with increased Hp (P-value = 0.190) or SAA (P-value = 0.197) between the five groups.

**Table 6.** Percentages of herbally supplemented dairy goats (100% = 12 goats) with acute phase proteins in excess of the reference intervals (according to Heller and Johns (12))

Group	Hp (>1.242 g/L)	SAA > 2.050 mg/L
7/20 g	0	0
7/40 g	0	25.0
9/20 g	0	8.3
9/40 g	8.3	16.7
CTR	16.7	25.0
Maximum likelihood G-test P-value	0.190	0.197

1 Hp – haptoglobin; SAA – serum amyloid A; 7/20 g and 7/40 g – groups supplemented with seven herbs at 20 or 40 g/animal/day; 9/20 g and 9/40 g – groups supplemented with nine herbs at 20 or 40 g/animal/day; CTR – control group

Spearman’s rank correlation coefficients between APPs and haematological and biochemistry parameters are presented in Table 7. Statistically significant (P-values ≤ 0.05 and ≤ 0.01) negative correlations between haematological parameters of blood plasma and the APP level were found between Hp and HGB, Hp and HCT and Hp and lymphocytes in blood from the 7/20 g group; between Hp and HGB and Hp and neutrophils in blood from the 9/20 g group; between SAA and monocytes in blood from the 9/40 g group; and between SAA and WBC and SAA and lymphocytes in blood from the CTR group. Positive correlations at the significance levels of P-value ≤ 0.05 and ≤ 0.01 were found between Hp and neutrophils in blood from the 7/20 g group; between Hp and HGB and Hp and MCHC in blood from the 9/40 g group; and between SAA and basophils in blood from the CTR group. Only one statistically significant negative correlation was found in the group of biochemical parameters, which was between Hp and HDL cholesterol in blood from the CTR group (P-value ≤ 0.05).

**Table 7.**
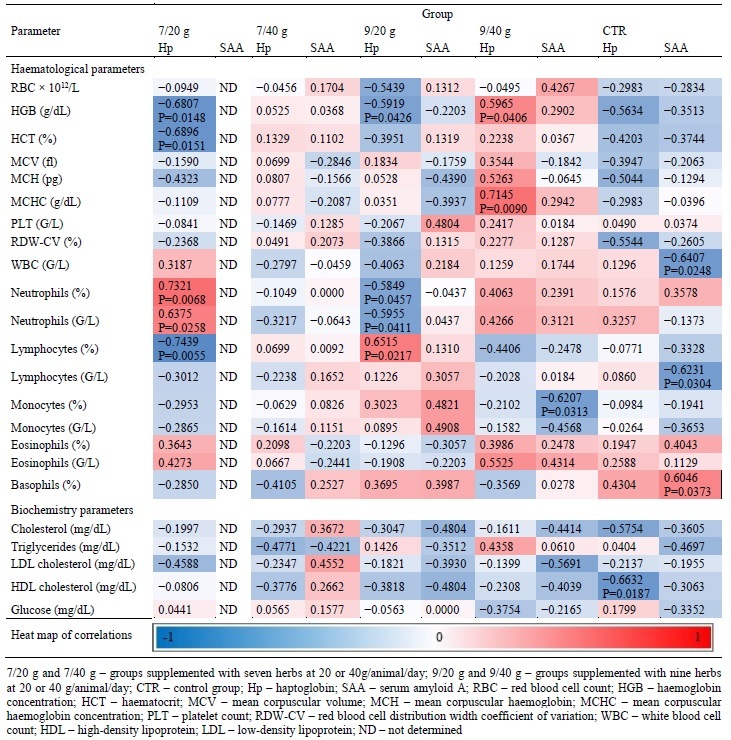
Spearman’s rank correlation coefficients between acute phase proteins and haematological and biochemistry parameters in blood samples from herbally supplemented dairy goats

## Discussion

The experimental herbal additives contained seven or nine different herb species. The large number of herbs used and their relatively ready availability make the preparations suitable for universal use. When designing the composition of the mixtures, the authors were guided by the content of bioactive substances and the typical properties of individual species of medicinal plants. The selected plants are thought to have a beneficial effect on animals, mainly in terms of improving digestion and metabolism. The proportion of herbs with such an effect was 50% in the seven-herb preparation and also 50% in the nine-herb preparation; the effect of stimulating milk production was one possessed by a 35% proportion of the seven-herb mixture and a 40% proportion of the nine-herb mixture, and bacteriostatic and anti-inflammatory effects were attributed to 55% of the composition of the seven-herb supplement and to 60% of the composition of the nine-herb counterpart.

The chemical composition of milk from all feeding groups of goats did not differ from the average values typical for goats’ milk. These, according to Siddiqui *et al*. (25), are a pH value of 5.05–6.72, TS of 12.07–12.97%, lactose content of 4.00–4.79%, protein content of 2.20–3.40%, fat content of 2.25–4.08%, casein content of 2.5% and FDP of -0.560. The freezing point ranged from –0.561 to –0.564°C and was consistent with the average range for goats’ milk (28).

The physiological concentration of urea in goats’ milk is not defined, and in reviewed investigations its concentration fluctuated between 217 and 624 mg/L (4). The urea concentrations obtained in our experiment were between 299 and 330 mg/L and within this range. The SCC in the experimental milk ranged from 534 to 560 × 10^3^ cells/mL, confirming that its cytological quality made it suitable for the production of high-quality dairy products, including fermented drinks. The SCC in goats’ milk is generally higher than in sheep’s and cows’ milk (13). According to the research of Kaskous *et al*. (13), the SCC in the milk of healthy goats fluctuates between 200 × 10^3^ and 1500 × 10^3^ cells/mL and is 764 × 10^3^ cells/mL on average.

In the current study, we did not find any effect of the herbal supplement on milk yield or FCM. In the experiment of Kholif *et al*. (15), a supplement of 10g/goat/day of lemongrass or rosemary herbs significantly increased the milk production of Damascus goats (P-value < 0.001). Moreover, goats obtaining these herbal supplements produced milk with higher protein, fat and dry matter than the control group (P-value < 0.001). Replacement of a concentrate feed mixture with *Moringa oleifera* leaf silage and *Chlorella vulgaris* microalgae mixture in the diet of Damascus goats improved milk yield and milk composition, particularly daily production of protein, fat and total solids (P-value < 0.05) (15).

All haematological data obtained in our study are within the physiological ranges for goats (1, 7). The HGB, RBC, WBC, MCV, MCH, MCHC and PLT were not influenced by diet. In the literature, these results were reported for clinically healthy goats to lie within the ranges of 9.05–9.65 g/dL, 11.82–12.98 T/L, 10.40–11.88 G/L, 19.81–20.24 fl, 7.54–7.70 pg, 37.10–38.75 g/dL and 279.17–330.00 G/L for HGB, RBC,WBC, MCV, MCH, MCHC and PLT, respectively (1, 2, 7, 20, 21). The normal RBC values observed in this study indicate that no goats were suffering from haemolytic anaemia and that erythrogenesis was not depressed. The haematological parameters of healthy goats, in particular RBC, HGB, HCT, MCV, MCH, MCHC, PLT and RDW-CV, show several differences and depend on the breed, sex and age of the goat (3). Arfuso *et al*.(3) analysed the blood of five native Italian goat breeds and found an effect of breed (P < 0.05) on RBC, HGB, HCT, MCV, MCHC and PLT. They also found an age and sex interaction affecting one parameter: two-year-old Messinese goats had more erythrocytes and higher HGB concentrations than older (>5 years) Messinese female goats (P < 0.05).

In the current study, we found an effect of herbal supplements on HCT in the peripheral blood of animals. The HCT measured in the blood samples of the group of goats receiving a mixture of 7 herbs in an amount of 20 g per day/animal was 22.68% and was the lowest among all nutritional groups (P-value < 0.05) (Table 4). There are examples in the scientific literature of feed additives that reduce the level of HCT in the blood of dairy goats (27). Giorgino *et al*. (9) found that adding citric and sorbic acid and thymol and vanillin to the nutritional ration of dairy goats reduced the HCT percentage on the 54^th^ (and last) day of the experiment (P-value < 0.05). In this experiment, all the morphological parameters of blood were within the physiological ranges of clinically healthy goats (1), confirming that supplementation with citric and sorbic organic acids and thymol and vanillin pure botanicals has no negative impact on the metabolic status of dairy goats. The addition of these components increased the fat level in milk (P-value = 0.04). A beneficial, statistically significant effect of the supplement on the milk coagulation index was also found (9).

Alongside erythrocytes and thrombocytes, leukocytes are one of the most important morphological elements of blood. Their functions include absorbing and digesting microorganisms, producing antibodies, and for certain of them, triggering the reaction of other appropriate leukocytes. There are five types of leukocytes: neutrophils (NEU) – polymorphonuclear leukocytes, eosinophils (EOS) – acidophils, basophils (BA), monocytes (MON) and lymphocytes (LYM). The sum leukocyte count (WBC) and the percentages of NEU, EOS, BA, MON and LYM determined in this study were similar for all feeding groups (P-value > 0.05) and were within the range of reference values given by Brooks *et al*. (5) for clinically healthy animals.

We also found a statistically significant decrease in the concentration of triglycerides in the blood of animals receiving herbal mixtures, especially in the 7/20 and 9/40 g groups This may have been due to the presence of phytochemicals in the herbal supplements that have the ability to reduce their synthesis and absorption (24). A similar decrease in triglyceride concentration was also found by Mohammadabadi *et. al*. (17), who fed dairy goats with fodder containing button mangrove (*Conocarpus erectus*) leaves and twigs.

Acute phase proteins constitute a large group of plasma proteins which mainly come from the liver in response to the pro-inflammatory cytokines IL-1, IL-6 and TNF-α. During an acute phase reaction (APR), the concentrations of APPs in the blood plasma rapidly increase, while in healthy animals, APPs are undetectable or present in negligible amounts, as noted by Heller and Johns (12). These researchers nominate APPs as useful indicators for detecting animals with subclinical infections, determining the prognosis of clinical infection, differentiating between viral and bacterial diseases, monitoring treatment and assessing vaccine effectiveness and stress conditions, and they consider Hp and SAA to be the major APPs in sheep and goats. The concentration of APPs in the blood serum of goats is not found in a specific range for a specific disease, but estimating it may help determine the animals’ health status (12). In our research Hp and SAA were within the RIs for healthy goats, *i.e*. 0.399–1.242 g/L and 0.435–2.050 mg/L, respectively (12).

The effect of a given amount of concentrate feed in goats’ diet on the APPs in blood plasma was studied by Wang *et al*. (29). They found statistically significant differences in Hg and SAA concentrations between the groups which received a high, medium or low amount of concentrate feed (P-value < 0.05). The levels of Hg and SAA determined in their studies were much higher than those found in ours, and were 1.46–1.85 g/L and 4.39– 5.48 mg/L, respectively (29). In the same study, a diet rich in rice improved the slaughter characteristics and meat quality of young Liuyang Black goats, but had a negative effect on gut microbiota and induced an APR, as the blood plasma SAA concentration was significantly increased (P-value = 0.010).

## Conclusion

The use of herbal supplements in the diet of dairy goats did not negatively affect their milk production, especially not milk yield or composition. In this study we found a positive effect of the administered multi-herbal mixtures on % fat concentration and fat : protein ratio in milk. Better results in this respect were achieved in the 7/20 g group. Time of test-day milking and milk sampling had highly significant effects on all studied milk parameters. Moreover, the feeding group × time of test-day milking and milk sampling interactions significantly affected milk yield and fat-corrected milk yield. The haematological and haematochemical parameters and APP plasma concentrations revealed no negative consequences of the supplementation of two multiherbal additives administered in the amounts of 20 or 40 g per animal per day. These findings encourage further research on herbal supplementation in the dairy goat diet.
